# Parental care in the Small Tree Finch *Camarhynchus parvulus* in relation to parasitism and environmental factors

**DOI:** 10.1111/ibi.12845

**Published:** 2020-05-27

**Authors:** Eileen Heyer, Arno Cimadom, Christian Wappl, Sabine Tebbich

**Affiliations:** ^1^ Department of Behavioural Biology University of Vienna Althanstraße 14 1090 Vienna Austria

**Keywords:** compensation hypothesis, Darwin’s finches, food provisioning, insecticidal treatment, *Philornis downsi*, rain, weed management

## Abstract

The parental food compensation hypothesis suggests that parents may compensate for the negative effects of parasites on chicks by increased food provisioning. However, this ability differs widely among host species and may also depend on ecological factors such as adverse weather conditions and habitat quality. Although weed management can improve habitat quality, management measures can bring about a temporary decrease in food availability and thus may reduce parents’ ability to provide their nestlings with enough energy. In our study we investigated the interaction of parasitism and weed management, and the influence of climate on feeding rates in a Darwin’s tree finch species, which is negatively impacted by two invasive species. The larvae of the invasive parasitic fly *Philornis downsi* ingest the blood and body tissues of tree finch nestlings, and the invasive Blackberry *Rubus niveus* affects one of the main habitats of Darwin’s tree finches. We compared parental food provisioning of the Small Tree Finch *Camarhynchus parvulus* in parasitized and parasite‐free nests in three different areas, which differed in invasive weed management (no management, short‐term and long‐term management). In a parasite reduction experiment, we investigated whether the Small Tree Finch increases food provisioning rates to nestlings when parasitized and whether this ability depends on weed management conditions and precipitation. Our results provide no evidence that Small Tree Finches can compensate with additional food provisioning when parasitized with *P. downsi*. However, we found an increase in male effort in the short‐term management area, which might indicate that males compensate for lower food quality with increased provisioning effort. Furthermore, parental food provisioning was lower during rainfall, which provides an explanation for the negative influence of rain on breeding success found in earlier studies. Like other Darwin’s finches, the Small Tree Finch seems to lack the ability to compensate for the negative effects of *P. downsi* parasitism, which is one explanation for why this invasive parasite has such a devastating effect on this host species.

Parasites impose strong selection on their hosts by impairing their growth, survival and/or reproduction (Price 1980, Loye & Zuk 1991, Lehmann 1993, Clayton & Moore 1997, Tschirren *et al.* 2009). This selective pressure will favour the evolution of effective counterstrategies in their host (Tschirren *et al.* 2009), including strategies to compensate for the damage caused by the parasite (Clayton & Moore 1997, Lemoine *et al.* 2011). Chicks of altricial birds have limited defences against parasites, which in turn selects for parental parasite control or compensation (Agrawal *et al.* 1999, Tschirren *et al.* 2009). One option to compensate for the energy loss of the chicks is to increase food provisioning (Johnson & Albrecht 1993, Tripet & Richner 1997, reviewed in Tschirren *et al.* 2009).

The parental food compensation hypothesis suggests that parents may increase their food provisioning rates to compensate for energy loss caused by parasites (Tripet & Richner 1997, Knutie *et al.* 2016). Empirical studies that have tested the parental food compensation hypothesis provide conflicting results, with supporting evidence in some species, e.g. Great Tits *Parus major* (Christe *et al.* 1996), Eurasian Blue Tits *Cyanistes caeruleus* (Tripet & Richner 1997, Hurtrez‐Boussès *et al.,* 1998) and the Galapagos Mockingbird *Mimus parvulus* (Knutie *et al.* 2016), but not in others, e.g. House Wrens *Troglodytes aedon* (Morrison & Johnson 2002), Darwin’s ground finches: Small Ground Finches *Geospiza fuliginosa*, Medium Ground Finches *Geospiza fortis* (Koop *et al.* 2013, O’Connor *et al.* 2014, Knutie *et al.* 2016), and Eurasian Penduline Tits *Remiz pendulinus* (Darolová *et al.* 1997).

One reason for these conflicting results could be that food provisioning depends on food availability, and thus when food is scarce, parents cannot compensate for the effect of parasites by increasing the rate at which chicks are fed. Food availability is influenced by environmental factors such as habitat quality and weather conditions (Mason 1944, Hollander *e* *t al.* 2015, McNew *et al.* 2019). Management measures that improve food availability may help parents to counteract energy loss caused by parasitism. However, management measures can sometimes also temporally decrease food availability and thus reduce parents’ ability to provide their nestlings with sufficient energy (Chiverton & Sotherton 1991, Moreby & Southway 1999, Boatman *et al.* 2004, Morris *et al.* 2005, Giuliano *et al.* 2018, Cimadom *et al.* 2019).

In the Galápagos Islands, long‐term habitat restoration programmes have been implemented to help recover the original habitat of Darwin’s finches endemic to the Galápagos Islands. One of the long‐term management programmes on Santa Cruz Island includes habitat restoration for arboreal Darwin’s finches (Woodpecker Finch *Camarhynchus pallidus*, Large Tree Finch *Camarhynchus psittacula*, Small Tree Finch *Camarhynchus parvulus* and the Green Warbler‐Finch *Certhidea olivacea*). This species group faces habitat changes due to invasive plant species and also is strongly affected by the invasive parasitic fly *Philornis downsi*. The parasitic larvae of this fly feed on blood and body tissues of the nestlings (Dudaniec & Kleindorfer 2006, Fessl *et al.* 2006b, O’Connor *et al.* 2010), causing significant mortality in Darwin’s finch chicks ranging from 16% to 100% in some years, with strong variation within and between host species (Cimadom *et al.* 2014, reviewed in Kleindorfer & Dudaniec 2016). Fitness loss of the hosts depends on parasite load, body size and brood size (Dudaniec *et al.* 2007). *P. downsi* abundance per chick decreases with increasing brood size, indicating a parasite dilution effect (Dudaniec *et al.* 2007). Compared with other Darwin’s finches, the arboreal finches are especially affected by *P. downsi* due to smaller brood sizes. A study by Cimadom *et al.*(2014) suggests that the high mortality in the Green Warbler‐Finch may also be explained by reduced food availability due to weed management. The main habitat of arboreal Darwin’s finches on Santa Cruz Island is a cloud forest (Dvorak *et al.* 2012) that has been heavily invaded by introduced plant species (Rentería *et al.* 2012). The Galápagos National Park Directorate (GNPD) controls invasive plants, leading to the temporary removal of the entire understorey (Filek *et* *al.* 2018). One year after this management measure, the breeding success of the Green Warbler‐Finch was significantly lower in areas that had been controlled recently than in areas without weed management (Cimadom *et al.* 2014). Cimadom *et al.* hypothesized that the control of invasive plants led to decreased food availability, thus reducing parents’ ability to provide their chicks with sufficient energy (Chiverton & Sotherton 1991, Moreby & Southway 1999, Boatman *et al.* 2004, Morris *et al.* 2005, Giuliano *et al.* 2018, Cimadom *et al.* 2019). An experimental study that aimed to test whether there is an interaction between habitat quality and parasitism revealed that Green Warbler‐Finches were able to compensate for parasitism when arthropod abundance was high but not when arthropod abundance was reduced due to control of invasive plants. In contrast, in the Small Tree Finch, breeding success was very low independent of weed management; only about 17% of the nests with chicks were successful, a rate considerably lower than in the Green Warbler‐Finch (53% of nests with chicks were successful, Cimadom *et al.* 2019). Breeding success in the Small Tree Finch only increased when nests were experimentally freed from parasites (Cimadom *et al.* 2019), indicating high virulence of *P. downsi* in this species. Moreover, Cimadom *et al.* (2014) found that nests of Green Warbler‐Finches and Small Tree Finches that experienced more intensive rainfall during chick‐rearing were less likely to produce fledglings. The authors hypothesized that parents are less active during intense rain and/or less efficient in foraging, which may lead to lower food provisioning rates. A negative influence of rain on food provisioning has been found in Great Tits (Radford *et al.* 2001).

Previous studies by Cimadom *et al.* (2014, 2019) could not explain why the breeding success of the Small Tree Finch was consistently extremely low between 2012 and 2014–2017 and why this species is particularly vulnerable to *P. downsi* parasitism. The focus of the current study was to investigate the role of food provisioning in relation to parasitism, weed management, arthropod abundance and weather condition, and thus provide a new angle to explain variation in fitness loss due to parasitism.

One explanation for why Cimadom *et al.*(2019) found an interaction between weed management and parasitism in the Green Warbler‐Finch but not in the Small Tree Finch is that the latter is more of a generalist, providing arthropods and plant food (mainly seeds of the dominant tree species *Scalesia pedunculata*) for their chicks (Tebbich *et al.* 2004, Filek *et al.* 2018). As the seeds of *S. pedunculata* are found in the canopy, which is not affected by weed management, a reduction in arthropod biomass is expected to affect the generalist feeder less than the specialist insectivorous species. Alternatively, arthropod food abundance or food quality are affected by weed management, but Small Tree Finches are able to compensate for it by adjusting food provisioning rates. Van Balen (2002) showed that there is a trade‐off between the quality and quantity of food provided by the parents, and that lower food quality (lower prey weight) results in higher feeding frequency.

In a parasite reduction experiment, we investigated whether Small Tree Finches increase food provisioning rates when parasitized, and whether the ability to compensate for parasitism depends on habitat quality in areas of different weed management regimens (no management, short‐term and long‐term management). We measured arthropod biomass in these three areas to establish whether food availability was still lower in the short‐term management area 2 years after the removal of the understorey. To test the influence of rain on food provisioning behaviour, we compared food provisioning rates during rainfall and in periods without rain.

We predicted that if Small Tree Finches compensate for parasitism, parents would show higher food provisioning rates in nests that are parasitized than in parasite‐free nests. If Small Tree Finches are affected by arthropod biomass, we would expect higher food provisioning rates in parasitized nests but only in areas with higher food availability. Additionally, we predicted that food provisioning rates would be reduced during rainfall.

## Methods

### Study site

The data for this study were collected during the breeding season of the Small Tree Finch from 31 January to 26 April 2017. The study was conducted in a *Scalesia* forest located at Los Gemelos in the highlands of Santa Cruz Island, Galápagos (0°37’34”S, 90°23’10”W). *Scalesia. pedunculata* is an endemic tree on the island and is the dominant tree species in the 24.5‐ha study site. Invasive plant species such as *Rubus niveus* are spreading in the understorey.

The GNPD is controlling invasive species expansion by manually cutting down invasive plants and subsequently applying herbicide. In the course of a larger experimental study on the impact of weed management on the ecosystem, we set up three management areas that differed in the degree and timing of invasive plant management (Cimadom *et al.* 2019):

1. ‘no management’ (8 ha), an area in which no control measures against invasive plant species have been taken;
2. ‘long‐term management’ (9.7 ha), an area in which the GNPD started rigorous weed management by cutting down the understorey and applying herbicides on a large scale in 2012 and thereafter followed up with localized herbicide applications on the invasive species’ regrowth;
3. ‘short‐term management’ (6.8 ha), an area in which the GNPD has been administering weed management since August 2014.

In addition, a 50 × 50‐m grid trail system was developed in the understorey vegetation in the no management area by National Park rangers (Cimadom *et al.* 2019).

### Nest monitoring and experimental treatment

Nest monitoring took place between January and April 2017 and followed the method of Cimadom *et al.* (2014). Data on breeding outcome and parasite abundance were part of the dataset published in Cimadom *et al.* (2019). Nests of the Small Tree Finch were monitored at regular intervals, using behavioural observations to determine the current breeding status: *building* – a male building a nest alone, *building with female* – already paired male and female building a nest together, *egg‐laying*, *incubation* and *feeding*. As soon as incubation began, a pole‐mounted endoscopic camera (dnt Findoo 3.6) was used to record clutch and brood size, as well as the age of the chicks. With the help of images of nestlings and dead chicks of known age, chick age in days could be estimated for nests where hatching date was unknown. In all instances, parents resumed parental care activities after we inspected the nests. We could therefore exclude nest desertion due to filming. Nests that produced at least one fledgling were defined as successful nests in accordance with Cimadom *et al.* (2014). Small Tree Finch chicks fledge at 14 ± 2 days (Cimadom *et al.* 2014). If chicks were more than 12 days old and the nest was found empty, chicks were considered to have fledged. Additionally, fledgling success was confirmed in all but one nest by observing the fledglings in the nest area. After nest failure or success, nests were collected and taken to the laboratory. Parasite intensity (as defined in Bush *et al.,* 1997) was determined as the total number of *P. downsi* larvae, pupae and empty puparia found per nest.

In 30 nests chosen randomly from a total of 61 nests, parasite infection was experimentally reduced through the injection of 10 mL of a 1% permethrin solution (PermectrinTM II) into the nest bottom with the help of a pole‐mounted syringe a maximum of 3 days before or after hatching. Permethrin is non‐toxic to birds and has been shown significantly to reduce parasite load and increase breeding success in the Small Ground Finch and the Medium Ground Finch (Fessl *et al.* 2006a, Koop *et al.* 2013, O’Connor *et al.* 2014, Knutie *et al.* 2016). After the treatment application, parents returned to the nest in all instances.

### Nest observations and food provisioning

For 61 Small Tree Finch nests, 121 nest observations of 1 h each were conducted during the nestling period. The observations were carried out between 06:00 and 14:35 h in random order. The frequency of feeding visits of females and males, males feeding females, and females feeding chicks directly after receiving food from the males was recorded. Feeding events that occurred completely inside the dome‐shaped nest could not be recorded. As the Small Tree Finch is a crop feeder, food was not visible, and the quality and quantity of the food could not be assessed. The expected feeding behaviour, i.e. storing food in the crop and leaning into the nest to regurgitate the food to the chicks, was observed. The duration of time females spent inside the nest was measured in minutes and was defined as the time the female spent completely inside the nest. As we did not have in‐nest cameras, we cannot specify the activities of the females inside the nests. However, it is likely that the females spent their time inside the nest brooding, preening, allopreening or just standing erect on the nest.

A feeding visit was defined as a parent feeding the chicks from outside the nest or half entering the nest to feed the chicks. Parental food provisioning was defined as the number of feeding visits to chicks per hour by both the male and the female. Male effort was defined as the sum of feeding visits of males, male feeding the female, and female feeding chicks directly after receiving food of the male per hour. Female effort was defined as the number of feeding visits of females per hour.

When possible, first observations were conducted at an early chick age (approximately 2 days old) and a second observation was carried out at chick age of 4 or 5 days. A second observation could not be conducted for all nests due to early nest failure. For 15 nests, we made a third 1‐h observation during rainfall. Rain and non‐rain nest observations occurred randomly in order.

### Sampling of arthropod biomass

Data on arthropod biomass were collected from a study on the impact of weed management on the breeding success of Darwin’s finches (Cimadom *et al.* 2019) from 2015 to 2017. For the present study, we re‐analysed the arthropod data from 2017.

We sampled arthropod biomass in the canopy, understorey and tree trunk moss, as Small Tree Finches mainly use these three micro‐habitats for foraging (Filek *et al.* 2018). Canopy samples were extracted by branch‐clipping, and arthropods within the moss were collected from the same trees from which the corresponding canopy samples were taken (for all details see Cimadom *et al.* 2019). The understorey samples were taken along a 5‐m‐long crosscut with a buffer of 1 m width in each direction, amounting to a total area of 10 m^2^. Arthropods discovered on vegetation of up to 1.7 m above ground were collected by visually searching or manual extraction, or by means of an aspirator for 15 min by one person. In each of the three study areas (no management, long‐term management and short‐term management), a canopy, understorey and moss sample were collected from 10 randomly selected sampling points in February and April of 2017. In total, 60 samples were taken.

All collected arthropods were identified to order, and their body length was measured (accuracy: ± 0.5 mm). We only considered Lepidoptera, Coleoptera, Orthoptera, Hymenoptera, Hemiptera, Diptera and Arachnida in analyses, as they represent the main food source for the Small Tree Finch (Filek *et al.* 2018). We calculated the dry weight for all sampled specimens of the relevant orders using specific length–weight regressions for each order (for details see Cimadom *et al.* 2019). Finally, we standardized the arthropod biomass per sampled canopy or moss plant material. Total arthropod biomass per sample was divided by the dry mass of the corresponding sampled plant material (mg arthropods/g plant material). As it was not feasible to collect the understorey vegetation, understorey samples were not standardized for the quantity of sampled vegetation.

### Statistical analyses

For all models, only observations of nests with chicks at the age of 6 days or younger were used and observations of nests with missing data were excluded. This resulted in a sample size of 59 nests and 100 observations of nests for the parental food provisioning model as well as the male effort model and the female duration inside the nest model. For the female effort model, the sample size was 59 nests and 99 observations of nests. For the breeding success model, we used 57 nests and 97 observations of nests. All statistical analyses were calculated with R, version 3.3.1 (R Core Team 2016), within R STUDIO, version 1.1.456 (R Studio Team 2016), using the packages *lme4* (Bates *e* *t al.* 2015) and *MuMin* (Barton 2016).

#### Parental effort

To analyse the relationship between parental food provisioning and influencing factors, we calculated a generalized linear mixed model (GLMM) with Poisson error structure and individual nest as a random effect (random intercept). For fixed effects, we used management area, treatment, the interaction of management area * treatment, age of chicks at observation, number of chicks and rain (yes/no). Additional GLMMs (family Poisson) with individual nest as a random effect (random intercept) were performed to analyse the effect of management area, treatment, the interaction of management area * treatment, age of chicks at observation, number of chicks and rain on male and female effort separately. We did not include an interaction term treatment * rain in the models as it made them less stable because of limited sample size of observations under rainy conditions.

To investigate the relationship between female duration inside the nest and influencing factors, we calculated a linear mixed model (LMM) with individual nest as a random effect (random intercept). As fixed effects, we used treatment, rain, age of chicks at observation and number of chicks.

For each of the above‐described models, a set of models containing no factors (null model), single factors or all possible combinations of factors was calculated. All models within a set were ranked according to their Akaike information criterion with adjustment for small sample size (AICc). Models with a delta‐AICc < 2.0 from the top ranked model were used to calculate model‐averaged estimates (full average), standard errors and 95% confidence intervals for each factor (Burnham & Anderson 2002). Model averaged predicted values for each set of observed values of the independent variables were used for graphical presentation.

#### Breeding success

To test how management area, *P. downsi* treatment and parental food provisioning influence breeding success, we first calculated a GLMM (Poisson error structure) with parental food provisioning as a dependent variable, where rain, age of chicks at observation and number of chicks were considered fixed effects and individual nest a random effect (random intercept). We then used the Pearson residuals of this model (feeding rate residuals) as a predictor in the breeding success model. For nests with more than one observation, we used the mean of the respective feeding rate residuals. For the breeding success (yes/no) model, we constructed a GLM (binomial family and logit link function) and used management area, treatment and feeding rate residuals as fixed effects. The significance of individual model terms was tested with Type II tests using the ANOVA procedure in the car package (Fox & Weisberg 2011).

#### *Philornis* abundance

To test whether the permethrin treatment reduced the parasite numbers in the nests and whether parasite abundance differed between management areas, we calculated a GLM with negative binomial error structure (because of overdispersion), with the number of *P. downsi* as the dependent variable and management area and treatment as fixed effects. We also included age of chicks at failure or fledgling as a co‐variable, as it was shown that parasite number increases with chick age (Cimadom *et al.* 2019). The significance of individual model terms was tested with Type II tests using the ANOVA procedure in the car package (Fox & Weisberg 2011).

#### Arthropod biomass

To test for differences in arthropod biomass among the areas, we calculated separate LMMs for the canopy, understorey and moss samples. We applied a log(*x* + 1) transformation to fulfil the criterion of normally distributed residuals. Management area was considered a fixed effect, and individual sampling points were entered as random effects (random intercept). *P*‐values were obtained by likelihood‐ratio tests of the full model against the null‐model without the effect in question.

## Results

### Parental food provisioning

**Figure 1:**
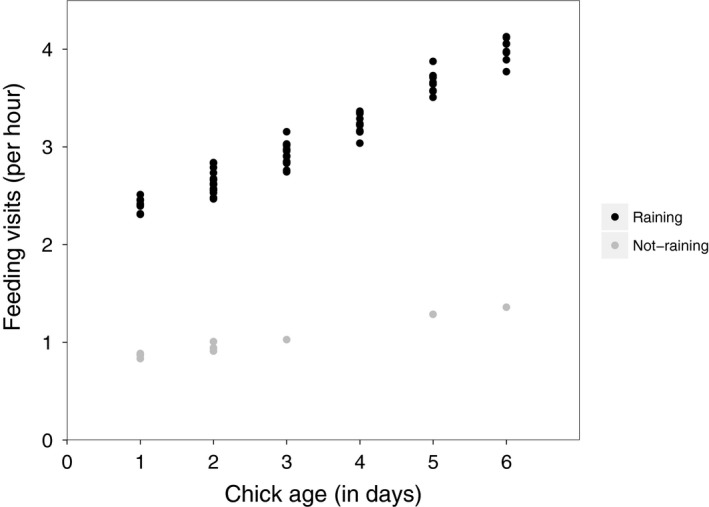
Parental food provisioning per hour dependent on chick age (in days) during dry hours (black) and rainy hours (grey, *n* = 100). Values represent model‐averaged predicted values of observed values of the independent variables.

**Figure 2:**
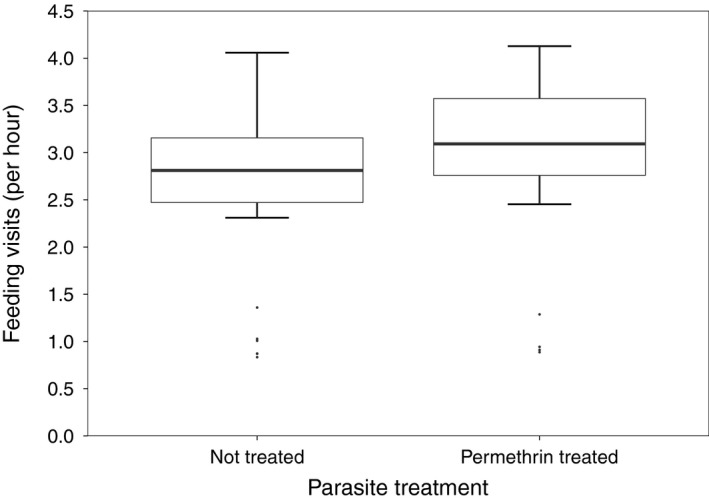
Parental food provisioning per hour at nests with permethrin treatment and nests without permethrin treatment (*n* = 100). Values represent model‐averaged predicted values of observed values of the independent variables.

All top models investigating parental food provisioning included the factors age of chicks and rain (relative variable importance = 1.00); none included the factor management area or the interaction term management area * treatment. Parental food provisioning increased with age of chicks (Table 1, Fig. 1) and was lower during rainy periods (Table 1, Fig. 1). The number of chicks being provisioned gained moderate importance in the models (relative variable importance = 0.36; Table 1). Treatment received only weak support (relative variable importance = 0.20; Table 1, Fig. 2).

**Table 1:** Outcomes from the model selection procedure using a subset of models with ΔAICc < 2.0 (see Table 2), showing relative variable importance, factor estimates ± standard errors (*b* ± se) and 95% confidence intervals (95% CI).

Dependent variable	Factors	Relative variable importance	*b* ± se	95% CI
Parental food provisioning	(Intercept)		0.66 ± 0.24	0.179–1.135
	Age of chicks	1.00	0.10 ± 0.04	0.027–0.181
	Rain	1.00	–1.02 ± 0.33	–1.668 to –0.372
	Number of chicks	0.36	0.04 ± 0.07	–0.100 to 0.178
	Treatment	0.20	0.02 ± 0.07	−0.113 to 0.153
Male effort	(Intercept)		0.68 ± 0.19	0.310–1.053
	Rain	1.00	−0.63 ± 0.29	−1.212 to −0.053
	Management area	0.85		
	Long‐term management – short‐term management		0.40 ± 0.28	−0.143 to 0.941
	Long‐term management – no management		0.20 ± 0.24	−0.270 to 0.661
	Short‐term management – no management		−0.20 ± 0.21	−0.610 to 0.204
	Treatment	0.39	−0.04 ± 0.20	−0.336 to 0.426
	Management area: treatment	0.23		
	Long‐term management: treated – short‐term management: treated		−0.17 ± 0.33	−0.822 to 0.492
	Long‐term management: treated – no management: treated		−0.14 ± 0.31	−0.749 to 0.473
	Short‐term management: treated – no management: treated		−0.20 ± 0.21	−0.333 to 0.387
	Age of chicks	0.11	−0.004 ± 0.02	−0.042 to 0.034
	Number of chicks	0.10	−0.005 ± 0.03	−0.067 to 0.057
Female effort	(Intercept)		−0.11 ± 0.32	−0.755 to 0.525
	Age of chicks	1.00	0.14 ± 0.05	0.033– 0.251
	Rain	1.00	−1.27 ± 0.51	−2.282 to − 0.252
	Number of chicks	0.28	0.04 ± 0.09	−0.132 to 0.212
	Management area	0.17		
	Long‐term management – short‐term management		−0.02 ± 0.10	−0.215 to 0.165
	Long‐term management – no management		0.04 ± 0.12	−0.195 to 0.270
	Short‐term management – no management		0.06 ± 0.16	−0.260 to 0.385
	Treatment	0.15	0.01 ± 0.07	−0.161 to 0.199
Female duration in nest	(Intercept)		44.90 ± 4.33	36.321– 53.474
	Age of chicks	1.00	−4.55 ± 0.92	−6.385 to − 2.714
	Treatment	1.00	−6.58 ± 2.92	−12.370 to −0.781
	Rain	0.31	1.68 ± 3.62	−5.461 to 8.823
	Number of chicks	0.19	−0.21 ± 0.93	−2.055 to 1.638

Only factors that were included in the model averaging procedure are shown in the table.

**Table 2:** Model selection procedure based on ΔAICc and Akaike weight (ω) for each calculated model.

Dependent variable	Fixed effects	AICc	Δ AICc	ω
Parental food provisioning	A + R	385.56	0.00	0.44
	A + R + N	385.95	0.39	0.36
	A + R + T	387.10	1.54	0.20
Male effort	R + Ma	339.07	0.00	0.25
	R + Ma + T + Ma:T	339.22	0.15	0.23
	R + Ma + T	340.07	1.00	0.15
	R	340.10	1.03	0.15
	R + Ma + A	340.66	1.59	0.11
	R + Ma + N	340.99	1.92	0.10
Female effort	A + R	308.38	0.00	0.40
	A + R + N	309.05	0.67	0.28
	A + R + Ma	310.09	1.71	0.17
	A + R + T	310.35	1.97	0.15
Female duration in nest	A + T	822.90	0.00	0.50
	A + T + R	823.85	0.95	0.31
	A + T + N	824.83	1.93	0.19

In the analysis, management area (Ma), treatment (T), rain observation (R), age of chicks (A) and number of chicks (N) were entered as fixed effects. Only models with a ΔAICc < 2.0 are shown for each analysis.

### Male effort

**Figure 3:**
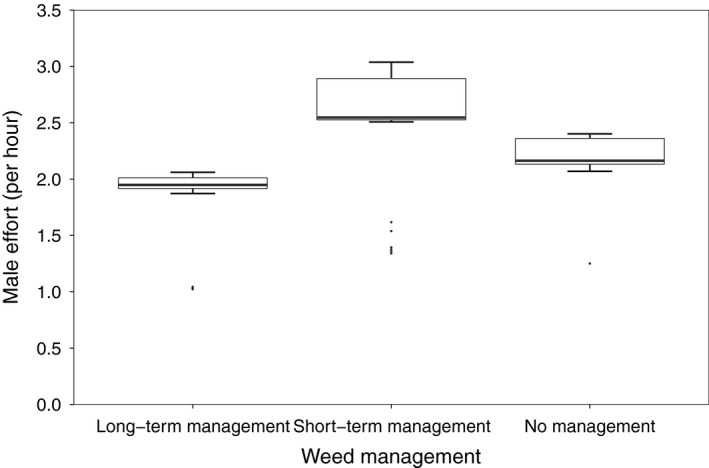
Male effort per hour dependent on the weed management (long‐term management, short‐term management, no management, *n* = 100). Values represent model‐averaged predicted values of observed values of the independent variables.

All top models investigating male effort included the factor rain (relative variable importance = 1.00). Male effort was lower during rainy periods (Table 1). Management area also showed strong importance (relative variable importance = 0.85). Male effort was higher in the short‐term management area than in the long‐term and no management areas (Table 1, Fig. 3). Treatment, the interaction of management area * treatment, age of chicks and number of chicks received very weak support in the models (relative variable importance = 0.39, 0.23, 0.11 and 0.10, respectively; Table 1).

### Female effort

All top models investigating female effort included the factors age of chicks and rain (relative variable importance = 1.00) and none of the models included the interaction term management area * treatment. Female effort increased with age of chicks and was reduced during rainy periods (Table 1). Number of chicks, treatment and management area were all of low importance in the models (relative variable importance = 0.28, 0.17 and 0.15, respectively; Table 1).

### Duration of females inside the nest

All top models investigating the duration of the female inside the nest included the factor age of chicks and treatment (relative variable importance = 1.00). The time females spent inside the nest decreased with age of the chicks and was higher in untreated nests (time spent in nests, mean ± se: untreated 30 ± 3 min; treated 23 ± 3 min; Table 1). Rain and number of chicks received very weak support in the models (relative variable importance = 0.31 and 0.19; Table 1).

### Nest success

Treatment had a significant influence on breeding success (χ^2^ = 6.05, *P* < 0.014), whereas management area (χ^2^ = 0.80, *P* = 0.669) and feeding rate residuals (χ^2^ = 0.18, *P* = 0.668) had no significant influence. Treated nests had significantly higher breeding success than untreated nests.

### Parasite treatment and management areas

Treated nests (median = 0, range = 0–59) had a significantly lower total number of *P. downsi* than untreated nests (median = 28, range = 0–86, χ^2^ = 9.62, df = 1, *P* = 0.002). Therefore, it can be assumed that the permethrin treatment was effective even when treatment was applied at different dates around the chick‐hatching date. There was no difference in parasite abundance between the three different management areas (no management: median = 36, range = 0–86, *n* = 9; short‐term management: median = 20.5, range = 0–58, *n* = 8; long‐term management: median = 30, range = 0–69, *n* = 12; χ^2^ = 5.05, df = 2, *P* = 0.08). Parasite abundance was independent of chick age at failure or fledging (χ^2^ = 0.01, df = 1, *P* = 0.91). The number of chicks did not differ between the three areas (Kruskal–Wallis test: *H* = 1.97, df = 2, *P* = 0.37, no management: median = 2, range = 1–3, *n* = 18; short‐term management: median = 2, range = 1–4, *n* = 22; long‐term management: median = 3, range = 1–4, *n* = 21).

### Arthropod biomass

**Figure 4:**
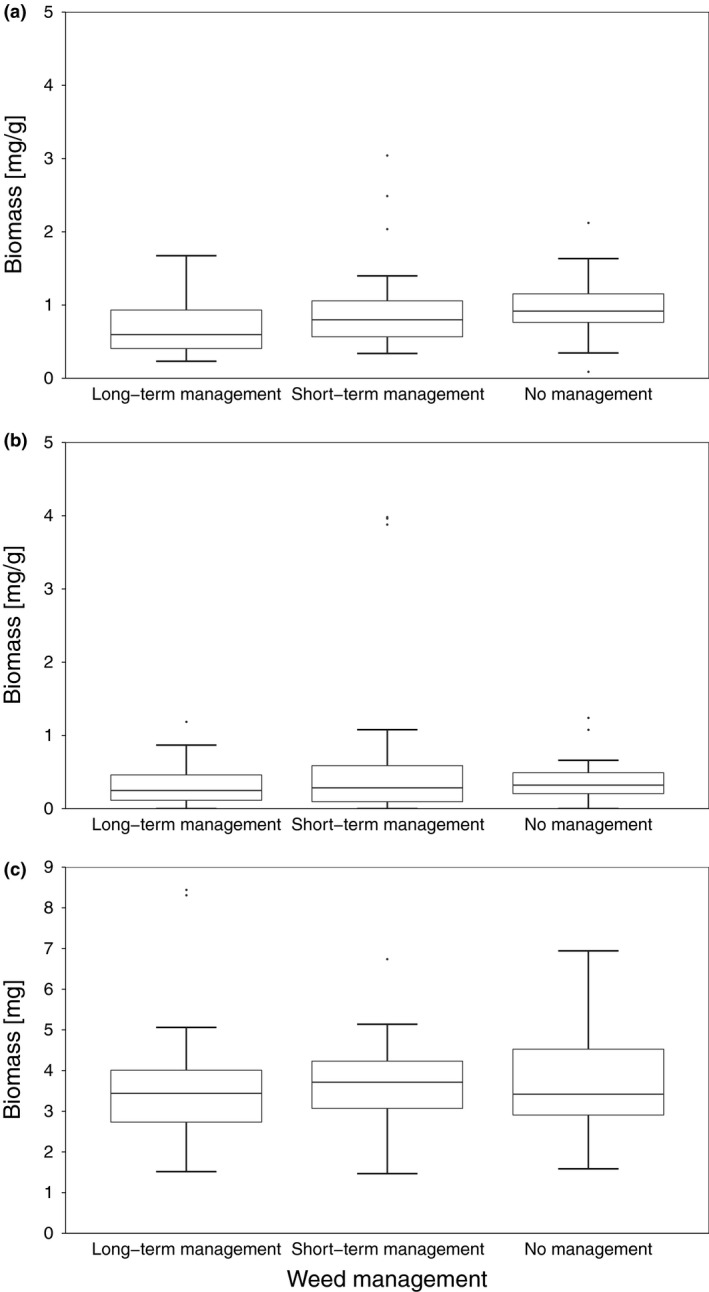
Arthropod biomass in three different weed management areas (long‐term management, short‐term management, no management) of three sampled strata: (a) the canopy (mg/g), (b) the moss layer (mg/g) and (c) the understorey (g). Given values of arthropod biomass were log (*x* + 1) transformed. Boundaries of the boxes represent 1st and 3rd quartiles, the black line within the boxes marks the median and whiskers extend from the median to the largest and lowest value within the 1.5 * IQR (interquartile range).

There was no significant difference in arthropod biomass among the three management areas in any of the three sampled strata (canopy: χ^2^ = 4.30, df = 2, *P* = 0.12; moss: χ^2^ = 3.20, df = 2, *P* = 0.20; understorey: χ^2^ = 0.03, df = 2, *P* = 0.98; Fig. 4).

## Discussion

### Effect of parasitism on parental food provisioning and brooding

We have not found any evidence that parents compensate with increased parental food provisioning rates when parasitized with *P. downsi*. Treatment reduced the intensity of parasitic flies in the nests; however, treatment had no effect on parental provisioning rates. This result is consistent with findings of similar studies on Medium Ground Finches and Small Ground Finches (O’Connor *et al.* 2014, Knutie *et al.* 2016), in which parasitism did not affect feeding frequencies of parents.

We did find that females spent a greater amount of time in parasitized nests. Fessl *et al.* (2006a) showed that parasitized chicks are developmentally delayed, which could have caused extended brooding, but we did not measure whether chicks were less developed in parasitized nests. Females could regard the inactivity of the chicks caused by energy loss due to parasites (Morrison & Johnson 2002) or begging intensity as an indicator for the developmental stage of the chicks. This result contrasts with studies on Galapagos Mockingbirds (Knutie *et al.* 2016) and Medium Ground Finches (Koop *et al.* 2013). In both latter species, females spent less time brooding their chicks in parasitized nests. Koop *et al.* (2013) revealed with the help of in‐nest cameras that female Medium Ground Finches attempted to avoid contact with the parasite by spending more time standing erect in the nest. We did not use in‐nest cameras and therefore cannot say if females spent the time brooding or standing erect in the nest.

### Effect of weed management and *Philornis* treatment on parental food provision

We tested the hypothesis that parents may not be able to increase food provisioning rates due to food limitation by comparing feeding frequencies in three areas with different weed management practices and their interaction with *Philornis* treatment. We found no difference in parental food provisioning among the three management areas and no interaction with *Philornis* treatment. One explanation for the above result is that we did not find a difference in arthropod biomass among the three different management areas. Weed management led to reduced arthropod biomass in areas with recent (< 2 years) weed management (Cimadom *et al.* 2019). In our study, initial weed management had been conducted more than 2 years before data collection, which could explain our negative result. However, we found increased male effort in the short‐term management area but no increase in breeding success. One explanation for these results is that in the short‐term management area, food quality was lower and males were able to compensate for it by increasing food provisioning rates. In a previous study we found that removal of the understorey resulted in lower arthropod species richness 1 year after treatment (P. Schmidt Yáñez diploma thesis, University of Göttingen), and it is possible that the effect on arthropod species composition is more enduring than the effect on arthropod abundance. For example, Great Tits compensate for lower food quality (lower prey weight) by increasing feeding frequency (Van Balen 2002). In the crop‐feeding Small Tree Finch, we were unable to assess the quantity and the quality of the food delivered by the parents. Using different methodological approaches such as stable isotope analysis of the chicks’ blood could help to detect differences in food quality. The relative variable importance of the interaction between weed management and *Philornis* treatment in the male effort model was low, and thus there is no indication that increased food provisioning rates compensate for parasitism.

Interestingly, the effect of the management area was not found in the female effort model. In the Black‐capped Chickadee *Poecile atricapillus*, males alone provide food in the earlier stage of the nestling period and females tend to brood the chicks (Smith 1997). Therefore, the effects of low food quality may be better reflected in the male effort than in the female effort. A study on Great Tits found that only males increased the feeding frequencies in parasitized nests, suggesting that males invest more in the current brood rather than in future reproduction compared with females (Christe *et al.* 1996).

### Effect of rain on parental food provisioning

In accordance with our prediction, we found that rain had a negative effect on the food provisioning frequency for both males and females. This contrasts with findings in Great Tits, where only females reduced their feeding rates during rain due to increased brooding activities, while male feeding rates stayed the same (Radford *et al.* 2001). We can think of two possible explanations for why both sexes of the Small Tree Finch reduced their feeding visits: (1) birds reduced their foraging behaviour during rain because of the direct effect of the rain or (2) they are still foraging, but they are less efficient because insect activity is reduced due to heavy rain. More detailed observations of foraging behaviour are needed to distinguish between these two potential explanations, but both lead to a decreased number of feeding events and can explain the negative effects of rain on breeding success found by Cimadom *et al.* (2014).

## Conclusions

Our results suggest that Small Tree Finches cannot compensate for parasitism but may be able to compensate for reduced food availability or quality. The increased effort of males in the short‐term management area indicates that although arthropod abundance recovers 2 years after management, species composition may not, and that removal of the understorey leads to a long‐term reduction in habitat quality. The management applied by the National Park is currently the only available method to combat the invasive *R. niveus* and conserve the *Scalesia* forest until biological control methods become available (Jäger *et al.* 2017, Cimadom *et al.* 2019). To reduce the detrimental impact on Small Tree Finch reproduction, we would suggest that invasive species management should be conducted sequentially at a smaller scale in order to preserve sufficient suitable breeding habitat.

## Author contributions

EH, AC and ST conceived and designed the study. EH, AC and CW conducted the fieldwork. EH and AC analysed the data. EH, AC and ST wrote the paper.

## Data Availability

The data that support the findings of this study are openly available in ‘Phaidra’ at https://phaidra.univie.ac.at/o:1078981.
